# Unkeito Suppresses RANKL-Mediated Osteoclastogenesis via the Blimp1–Bcl6 and NF-κB Signaling Pathways and Enhancing Osteoclast Apoptosis

**DOI:** 10.3390/ijms23147814

**Published:** 2022-07-15

**Authors:** Ke Fang, Yuki Murakami, Seiji Kanda, Takaki Shimono, Anh Tuan Dang, Mitsuaki Ono, Toshimasa Nishiyama

**Affiliations:** 1 Department of Hygiene and Public Health, Kansai Medical University, 2-5-1 Shin-machi, Hirakata 573-1010, Japan; houka@hirakata.kmu.ac.jp (K.F.); murakamy@hirakata.kmu.ac.jp (Y.M.); shimonot@hirakata.kmu.ac.jp (T.S.); tnishi@hirakata.kmu.ac.jp (T.N.); 2 Regenerative Research Center for Intractable Diseases, Kansai Medical University, 2-5-1 Shin-machi, Hirakata 573-1010, Japan; 3 Department of Molecular Biology and Biochemistry, Okayama University Graduate School of Medicine, Dentistry and Pharmaceutical Sciences, Okayama 700-8558, Japan; ppjj5xuj@s.okayama-u.ac.jp (A.T.D.); mitsuaki@md.okayama-u.ac.jp (M.O.); 4 Department of Oral Rehabilitation and Regenerative Medicine, Okayama University Graduate School of Medicine, Dentistry and Pharmaceutical Sciences, Okayama 700-8558, Japan; 5 Department of Oral Rehabilitation and Implantology, Okayama University Hospital, Okayama 700-8558, Japan

**Keywords:** osteoporosis, postmenopause, NFATc1, p65, caspase-3, Unkeito

## Abstract

Osteoporosis is a common bone disease, particularly in menopausal women. Herein, we screened four Kampo medicines (Unkeito (UKT), Kamishoyosan (KSS), Kamikihito (KKT), and Ninjinyoeito (NYT)), frequently used to treat menopausal syndromes, for their effects on receptor activator of nuclear factor-kappaB ligand (RANKL)-induced osteoclast differentiation in RAW 264 cells. Considering that UKT exhibited the most potent effect, we examined its effect on RANKL-induced osteoclastogenesis, the induction of osteoclast apoptosis, and the mechanisms underlying its effects. UKT inhibits RANKL-induced osteoclast differentiation in the early stage and decreases osteoclast-related genes, including tartrate-resistant acid phosphatase (*Trap*), dendritic cell-specific transmembrane protein (*Dcstamp*), matrix metalloproteinase-9 (*Mmp9*), and cathepsin K (*Ctsk*). Specifically, UKT inhibits the nuclear factor of activated T cells 1 (NFATc1), which is essential for osteoclastogenesis. UKT increases Bcl6, which antagonizes NFATc1 and Dc-stamp, thereby blocking the progression of osteoclasts to maturation. UKT also decreased nuclear translocation by downregulating the activity of p65/NF-κB. In addition, UKT enhances mononuclear osteoclast apoptosis via activation of caspase-3. Herein, we demonstrate that UKT suppresses RANKL-mediated osteoclastogenesis via the Blimp1–Bcl6 and NF-κB signaling pathways and enhances mononuclear osteoclast apoptosis. Furthermore, UKT prevents bone loss in OVX mice. Thus, UKT might be a potential therapeutic agent for postmenopausal osteoporosis.

## 1. Introduction

Osteoporosis is a clinically intractable disease that often causes fractures. In 2000, 9.0 million people were affected with osteoporotic fractures, with 5.8 million total disability-adjusted life years (DALYs) lost, of which 51% were fractures that occurred in Europe and the Americas [1]. Most cases occur in postmenopausal women owing to homeostasis imbalance from the dramatic decrease in estrogen levels, which accelerates the activation of osteoclasts for bone resorption [2]. As osteoclasts play a major role in bone resorption, they can destroy the bone matrix, which is the direct cause of osteoporosis [3]. Thus, understanding the effects of the available therapeutic agents on osteoclasts is important for the effective treatment of osteoporosis.

The mouse monocyte RAW 264.7 cell line has been widely used to study the mechanism of osteoclastogenesis. RAW 264.7 cells differentiate into osteoclasts by the stimulation of receptor activator of nuclear factor-κB (NF-κB) ligand (RANKL) [4]. It has been demonstrated that RANKL-induced osteoclast differentiation in these cells is regulated by multiple pathways. Binding of RANKL to its receptor, RANK, promotes osteoclast differentiation through the recruitment of tumor necrosis factor (TNF) receptor-associated factor 6 (TRAF6), subsequently activating downstream signaling pathways, namely the NF-κB signal [5,6,7] and mitogen-activated protein kinase (MAPK) pathway [8,9,10]. RANKL-mediated up-regulation of the NF-κB signal and MAPK pathway activates the essential transcription factor of nuclear factor of activated T cells 1 (NFATc1), and activated NFATc1 induces expression of osteoclastic molecules essential for osteoclast differentiation and function, such as tartrate-resistant acid phosphatase (TRAP), which is a cytochemical marker for both mononuclear and mature osteoclasts, dendritic cell-specific transmembrane protein (DC-STAMP) to promote cell-cell fusion, and MMP-9 and cathepsin K (CTSK) to degrade bone matrix and collagen proteins [11,12,13]. On the other hand, recently, novel transcriptional repressors, B lymphocyte-induced maturation protein-1 (Blimp1) and B cell lymphoma 6 (Bcl6), controlling osteoclastogenesis were identified. Bcl-6 negatively regulates expression of osteoclast-specific genes, which are NFATc1 targets. It also has been shown that Blimp1 binds to the Bcl6 promotor and suppresses its expression [14]. Therefore, inhibition of NFATc1 is an initial approach to prevent osteoclastogenesis. In addition to inhibiting osteoclast differentiation, the induction of osteoclast apoptosis is another approach to prevent pathological bone resorption. It has been reported that calcitonin induces osteoclast apoptosis and increases its efficacy in an in vivo model of osteoporosis [15].

Kampo medicines are compound granules extracted from herbal drugs originating from classical or traditional Chinese medicine, which are frequently used in Japan due to their therapeutic efficacy and minimal side effects despite long-term use. In this study, we screened four Kampo medicines, namely, Unkeito (UKT), Kamishoyosan (KSS), Kamikihito (KKT), and Ninjinyoeito (NYT), which are clinically used in Japan to treat menopausal women with various symptoms.

Herein, we investigated their effectiveness and whether they have any effect on osteoclast differentiation. We also sought to understand the molecular mechanism underlying the action of the most effective Kampo medicine by studying key signaling pathways.

## 2. Results

### 2.1. UKT, KSS, KKT, and NYT Suppress Osteoclast Differentiation Induced by RANKL, Especially UKT

**Figure 1:**
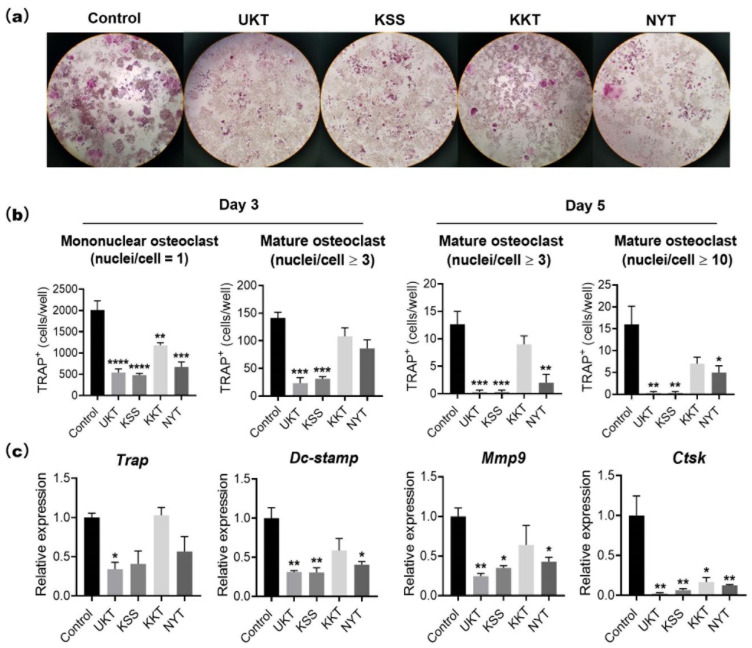
Unkeito (UKT), Kamishoyosan (KSS), Kamikihito (KKT), and Ninjinyoeito (NYT) decrease tartaric acid resistant phosphatase (TRAP)-positive cells at the different stages of osteoclast differentiation. (**a**) TRAP staining for osteoclasts on days 5 (Magnification: ×100); (**b**) number of TRAP-positive mononuclear osteoclasts (nuclei/cell = 1) and mature osteoclasts (nuclei/cell ≥ 3 or 10) on days 3 and 5 (*n* = 3); (**c**) the expression levels of osteoclast-related gene markers *Trap*, *Dcstamp*, *Mmp9*, and *Ctsk* normalized to that of *Gapdh*. Error bars represent ± standard error of the mean. * *p* < 0.05; ** *p* < 0.01; *** *p* < 0.005; **** *p* < 0.0001, versus the control groups.

We first examined the viability of RAW 264 cells after treatment with UKT, KSS, KKT, and NYT at low to high concentrations (50–500 µg/mL). No cytotoxicity was observed following KSS, KKT, or NYT treatment. However, the viability of RAW 264 cells decreased at 400 µg/mL and was significantly reduced to approximately 50% at 500 µg/mL in the UKT group compared to that of the control (Figure S1). To avoid cytotoxicity, 300 µg/mL was set as the maximum concentration. Next, we examined TRAP-positive osteoclasts induced by RANKL on days 3 and 5 with the concentration of 300 µg/mL (Figure 1a) by counting the number of TRAP-positive mononuclear osteoclasts and mature osteoclasts (Figure 1b). On day 3, corresponding to after differentiation, mononuclear (nuclei/cell = 1) osteoclasts were significantly reduced in all four treatment groups, and mature osteoclasts (nuclei/cell ≥ 3) were significantly reduced in the UKT and KSS groups in comparison to the control. On day 5, mature osteoclasts (nuclei/cell ≥ 3) were significantly reduced by UKT, KSS, and NYT; however, no significant difference was found in the KKT group compared with the control. Furthermore, the number of osteoclast giant cells (nuclei/cell ≥ 10) was significantly reduced by NYT compared to the control. In fact, no giant cells were detected in the UKT and KSS groups. These results suggest that the four compounds can suppress RANKL-induced mononuclear osteoclasts during early stages of differentiation, and that UKT, KSS, and NYT can inhibit the formation of mature osteoclasts, with UKT and KSS resulting in higher inhibition.

To further verify the impact of the four compounds on the stages of RANKL-induced osteoclast differentiation, the expression of osteoclast-specific genes during the various stages of differentiation was evaluated (Figure 1c). The level of *Trap* expression in the UKT group was significantly lower than that of the control on day 5 following RANKL induction. Dc-stamp is recognized as an intercellular fusion factor essential for the formation of mature osteoclasts [10]. The expression of *Dcstamp* and *Mmp9* was significantly decreased by UKT, KSS, and NYT compared to that of the control. Additionally, *Ctsk* expression was significantly decreased by all compounds compared to the control. These results imply that the four compounds could suppress osteoclast differentiation, with UKT determined to be the most effective of the four. Thus, UKT was assessed in all additional analyses.

### 2.2. UKT Activates the Suppression of NFATc1 at the Early Stage of Osteoclast Differentiation Induced by RANKL

**Figure 2:**
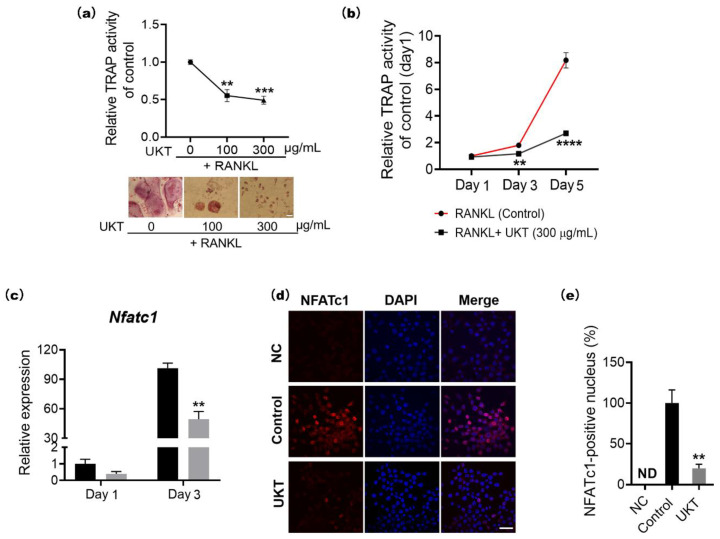
Unkeito (UKT) affects tartaric acid-resistant phosphatase (TRAP) activity in a concentration-dependent manner and at early stage of osteoclast differentiation. (**a**) RAW 264 cells treated with sRANKL (100 ng/mL) with or without UKT (100 and 300 µg/mL) for five days (*n* = 4; scale bar = 25 µm); (**b**) RAW 264 cells (1 × 10^3^ cells/96-well) treated with sRANKL (100 ng/mL) with or without UKT at 300 µg/mL for one, three, or five days (*n* = 4); (**c**) mRNA expression level of *Nfatc1* normalized to that of *Gapdh*; (**d**) protein abundance of NFATc1 72 h after RANKL stimulation in the presence or absence of UKT (*n* = 3; scale bar = 20 µm); (**e**) intra-nuclear abundance of NFATc1. Error bars represent ± standard error of the mean. ** *p* < 0.01; *** *p* < 0.005; **** *p* < 0.0001, versus control groups on the same day. Black box: control; gray box: UKT; NC: negative control; ND: not detected.

To determine whether UKT has effects on RANKL-induced osteoclast differentiation in a concentration-dependent manner, TRAP activity was measured following treatment with different concentrations (100 and 300 µg/mL) of UKT. Activity was significantly reduced following treatment with either concentration compared to that of the control, although the effect of the 300 µg/mL concentration was more obvious than the 100 µg/mL (Figure 2a). To examine the effects of UKT on different stages of osteoclast differentiation, cells were cultured with or without 300 µg/mL after induction by RANKL for one, three, and five days. UKT significantly decreased TRAP activity from day 3 until day 5 compared with the control (Figure 2b). To better understand the mechanism underlying the inhibitory effect of UKT on osteoclastogenesis, the expression of NFATc1, an essential osteoclastogenesis transcription factor, was examined using quantitative polymerase chain reaction (qPCR). UKT significantly decreased *Nfatc1* expression compared to that of the control on day 3 after RANKL induction (Figure 2c). Immunocytochemistry staining further revealed a significantly lower abundance of NFATc1 within cell nuclei following treatment with UKT (Figure 2d,e). Thus, UKT decreased both the mRNA and protein levels of NFATc1. The results suggest that UKT may affect the early stage of osteoclast differentiation.

### 2.3. UKT Activates the Suppression of NFATc1 by Overexpressing Bcl6 through the Blimp1–Bcl6 Axis

**Figure 3:**
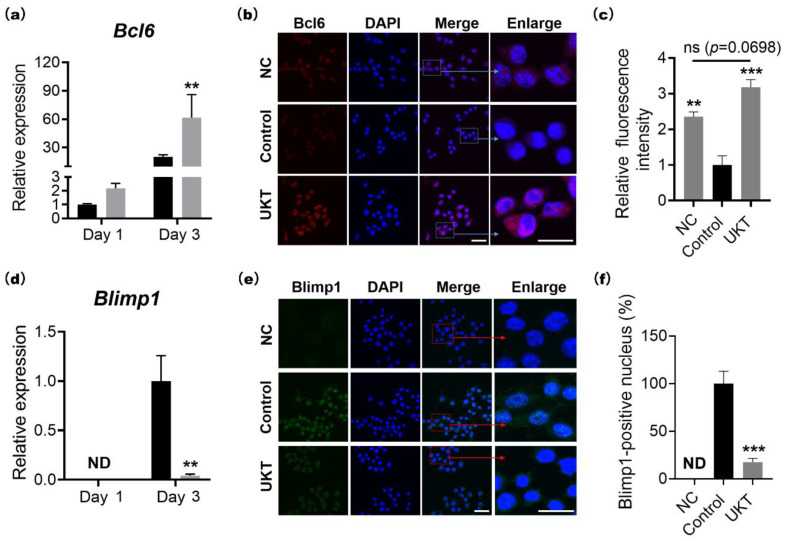
Unkeito (UKT) suppresses nuclear factor of activated T cells 1 (NFATc1) through the Blimp1–Bcl6 axis by increasing Bcl6 expression. (**a**) mRNA expression level of *Bcl6* normalized to that of *Gapdh*; (**b**) abundance of Bcl6 72 h after RANKL stimulation in the presence or absence of UKT (*n* = 3; scale bar = 10 µm, 2 µm); (**c**) relative fluorescence intensity of Bcl6; (**d**) mRNA expression level of *Blimp1* normalized to that of *Gapdh*; (**e**) protein level of Blimp1 72 h after RANKL stimulation in the presence or absence of UKT (*n* = 3; scale bar = 10 µm, 2 µm); (**f**) intra-nuclear level of Blimp1. Error bars represent ± standard error of the mean. ** *p* < 0.01, *** *p* < 0.005, versus control groups on the same day. Black box: control; gray box: UKT; NC: negative control; ND: not detected.

As Bcl6 is a direct regulator of NFATc1, its expression was analyzed via qPCR and immunocytochemical staining. The results showed that the mRNA expression levels of Bcl6 were significantly increased by UKT compared to the control on day 3 after induction by RANKL (Figure 3a). To examine the protein activation of Bcl6, immunocytochemistry staining was performed. In the control group, the protein expression of Bcl6 was almost silent after 72 h of RANKL stimulation; in contrast, in the UKT group, the protein expression of Bcl6 was effectively activated and higher than that of the negative control (Figure 3b,c). This indicates that UKT induces Bcl6 overexpression during the suppression of osteoclastogenesis. Additionally, the mRNA expression of *Blimp1*, an upstream regulator of *Bcl6*, was correspondingly decreased (Figure 3d). Immunocytochemical staining also showed that the abundance of Blimp1 was significantly decreased in the nucleus after treatment with UKT (Figure 3e,f). Therefore, UKT reduced the mRNA and protein levels of Blimp1. Overall, our results suggest that UKT suppresses NFATc1 by overexpressing Bcl6 in the negative pathway through the Blimp1–Bcl6 axis.

### 2.4. UKT Inhibits NF-κB Signaling Pathway Induced by RANKL

**Figure 4:**
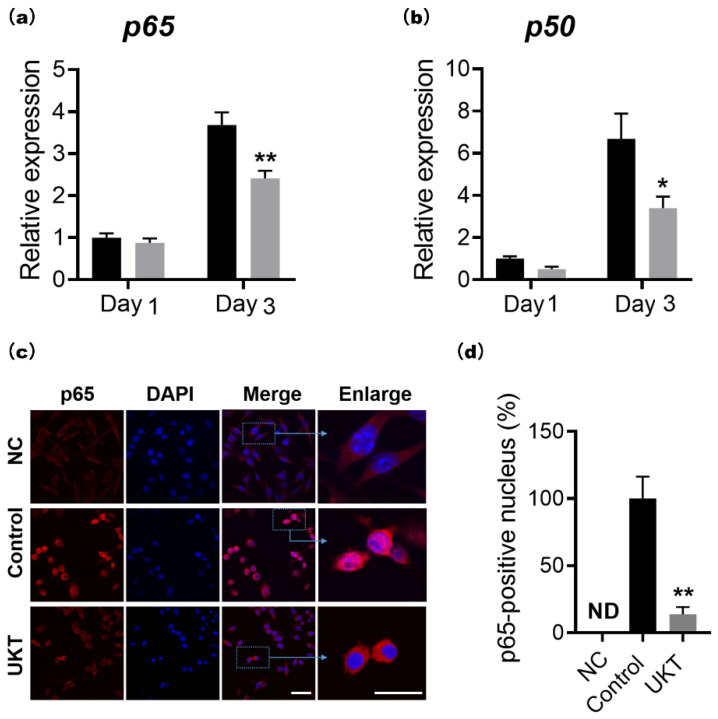
Unkeito (UKT) reduces p65 expression in the NF-κB signaling pathway. Expression of (**a**) *p65* and (**b**) *p50* normalized to that of *Gapdh*; (**c**) p65 localization 72 h after RANKL stimulation in the presence or absence of UKT (*n* = 3; scale bar = 10 µm, 2 µm); (**d**) p65 abundance in the nucleus. Error bars represent ± standard error of the mean. * *p* < 0.05; ** *p* < 0.01, versus control groups on the same day. Black box: control; gray box: UKT; NC: negative control; ND: not detected.

The NF-κB signaling pathway is one of the NFATc1 regulatory pathways. To determine whether UKT influences the NF-κB signaling pathway, the expression of *p65* and *p50* was evaluated via qPCR. The expression of both *p65* and *p50* was significantly lower following UKT treatment compared to the control on day 3 following RANKL induction (Figure 4a). To confirm the nuclear translocation of p65, immunocytochemistry staining was performed. In the UKT group, most p65 was within the cytoplasm, not translocated to the nucleus, compared to that by the control on day 3 after RANKL induction (Figure 4b,c). Collectively, these results indicate that UKT has regulatory effects on p65, thereby suppressing NFATc1 expression via the NF-κB signaling pathway. 

### 2.5. UKT Enhances Apoptosis of Mononuclear Osteoclasts

**Figure 5:**
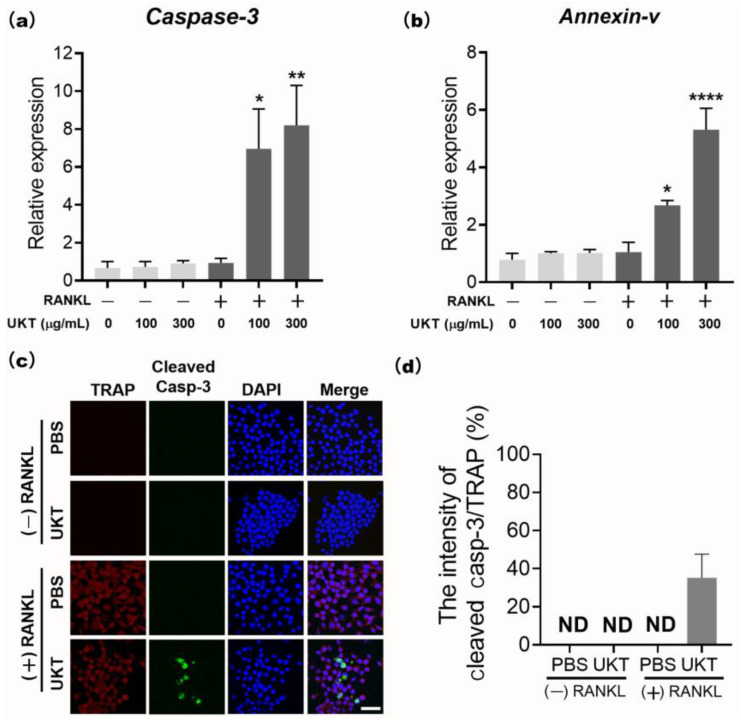
Unkeito (UKT) induces mononuclear osteoclast apoptosis via caspase-3 activation. (**a**) *caspase-3* and (**b**) *annexin-v* mRNA expression in the early stage of apoptosis, normalized to that of *Gapdh*; (**c**) cleaved caspase-3 abundance detected via immunocytochemistry (*n* = 3; scale bar = 10 µm); (**d**) the percentages of cleaved caspase-3 positive in total of tartaric acid resistant phosphatase (TRAP) -positive cells. Error bars represent ± standard error of the mean. * *p* < 0.05; ** *p* < 0.01, **** *p* < 0.0001, versus negative control groups (PBS without RANKL); ND: not detected.

Additionally, we examined whether UKT impacts osteoclasts via induction of apoptosis. The mRNA expression of the most sensitive indicators, *caspase-3* and *annexin-v*, in the early stage of apoptosis, was significantly increased following UKT treatment after 24 h in a concentration-dependent manner, compared to the negative controls (Figure 5a,b). Cleaved caspase-3, an activated form of caspase-3 critical to apoptosis, was evaluated by immunocytochemistry. Figure 5c,d show that cleaved caspase-3 was present in the TRAP-positive mononuclear osteoclasts following UKT treatment after 6 h, but no cleaved caspase-3 was found in RAW 264 cells, suggesting that apoptosis of mononuclear osteoclasts may result following induction of caspase-3 activation by UKT, thereby further preventing osteoclast maturation. These results demonstrate that UKT can enhance apoptosis of mononuclear osteoclasts induced by RANKL.

### 2.6. Oral Administration of UKT Prevents Bone Loss in Ovariectomy (OVX)-Mice

**Figure 6:**
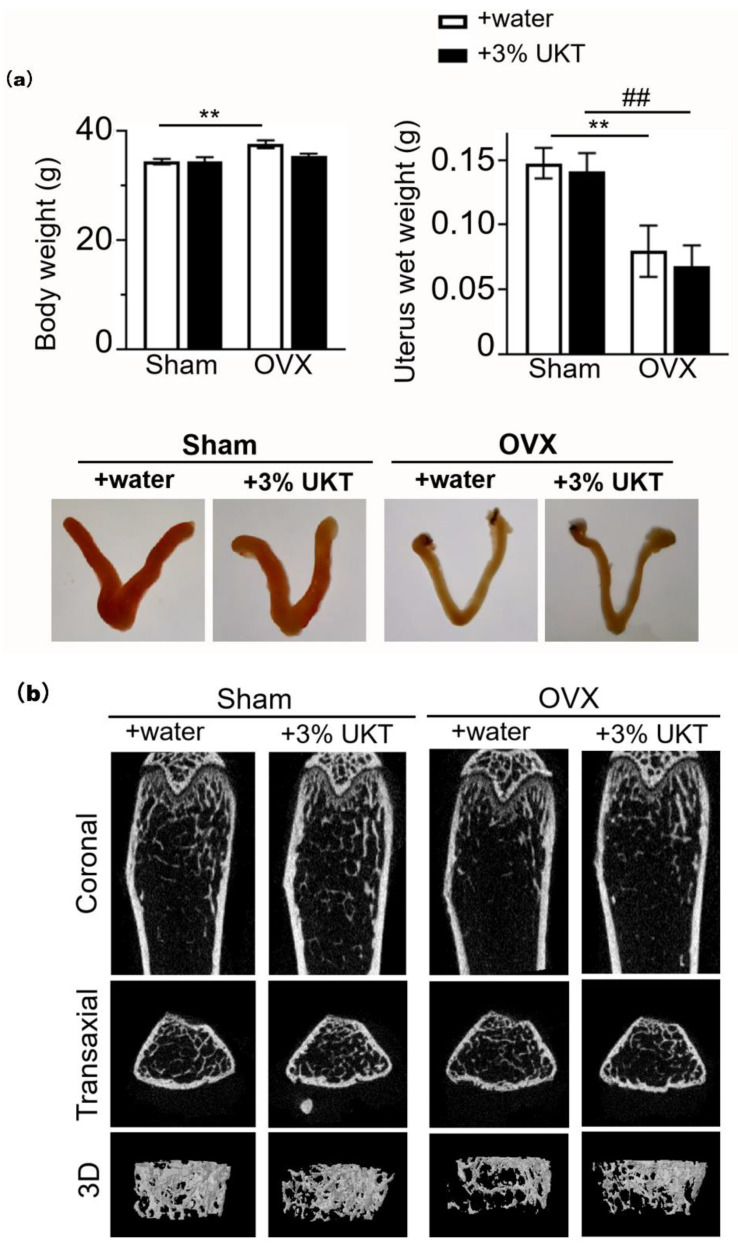

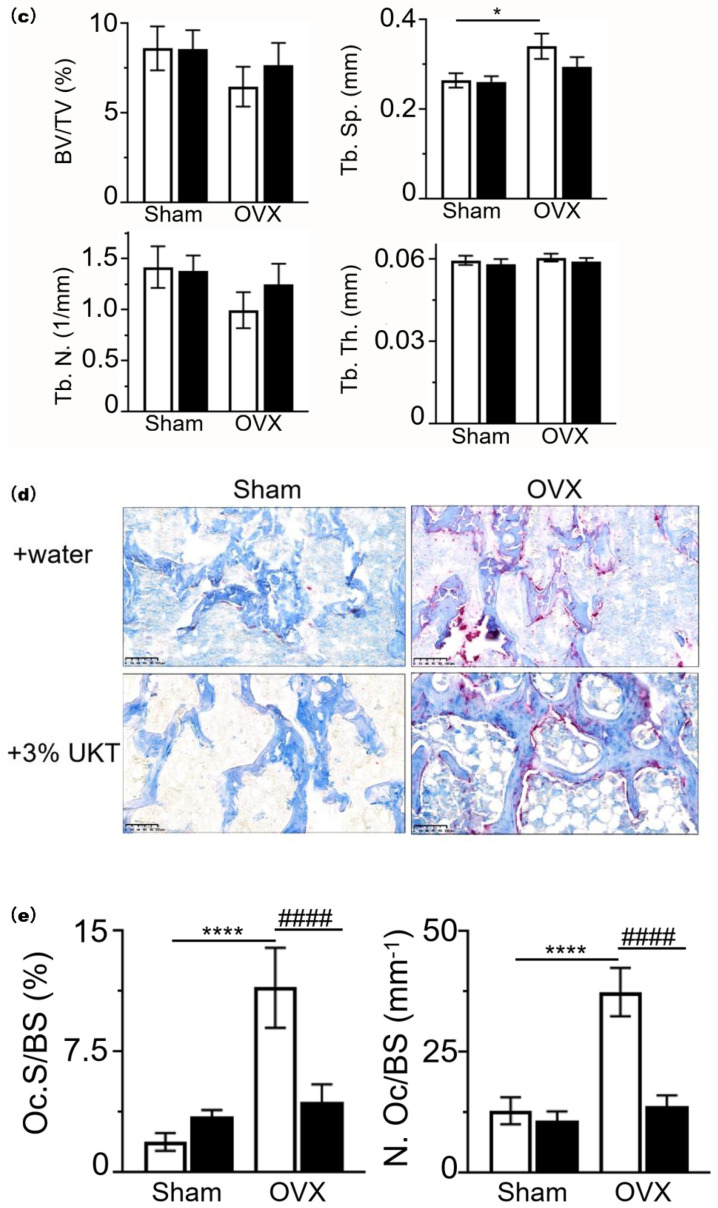
Oral administration of Unkeito (UKT) prevents bone loss in ovariectomy (OVX) mice. Eight-week-old CD-1 mice were daily administrated UKT in drinking water for 28 consecutive days, following sham or OVX operation (sham + water, *n* = 11; sham + 3% UKT, *n* = 11; OVX + water, *n* = 11; OVX + 3% UKT, *n* = 11). Open bar represents mice with water, while closed bar represents mice treated with 3% UKT. (**a**) Average of body weight and uterus wet weight on day 28 after surgery. (**b**) Images of the distal femur from micro-computed tomography analysis and quantitative data of BV/TV (%), Tb.Sp (mm), Tb.N (1/mm), and Tb.Th (mm) are shown in (**c**). (**d**) Typical pictures of the distal femur from aniline blue and tartrate-resistant acid phosphatase stains, and quantitative data of Oc.S/BS (%) and N.Oc/BS (mm^−1^) are shown in (**e**). Two-way ANOVA was used, followed by Tukey’s multiple comparison tests. Data are expressed as means ± standard error of mean for each group. * *p* < 0.05, ** *p* < 0.01, **** *p* < 0.0001 vs. sham-operated mice with water. ## *p* < 0.01, #### *p* < 0.0001 vs. OVX-operated mice with water. Bar = 100 µm. BV/TV, bone volume/tissue volume (bone volume fraction); Tb.Sp, trabecular separation; Tb.N, trabecular number; Tb.Th, trabecular thickness; Oc.S/BS, extent of osteoclast surface per bone surface; N.Oc/BS, number of osteoclasts per bone surface.

Mice were given drinking water containing 3% UKT following sham or OVX operation for 28 consecutive days. There were no significant changes in daily food and drinking water intake between all groups (Figure S2a). The daily intake of UKT was 6.4 g/kg/day for 28 consecutive days, considering the average daily intake of drinking water with 3% UKT and body weight (Figure 6a and Figure S2a). The human equivalent dose calculated based on the body surface was 0.55 g/kg/day [16], while the experimental dose was approximately 3–4 times higher than the clinically relevant dose in humans. Although OVX led to a markedly increased body weight and decreased uterus wet weight 28 days after the surgery, they showed no significant differences by UKT treatment (Figure 6a). Under these conditions, distal femoral trabecular bone volume trended toward reduction in the OVX mice with normal drinking water compared to sham mice (Figure 6b,c). UKT treatment considerably prevented trabecular bone loss in OVX mice femur (Figure 6b,c). Data analysis clearly showed that oral supplementation of UKT markedly inhibited the bone loss in OVX mice, and especially trabecular separation (Tb.Sp) was significantly improved by UTK treatment in OVX mice (Figure 6c).

Next, we conducted a histomorphometric analysis of the femur to define the cellular basis underlying the protective effect of UKT on bone loss. The number of TRAP-positive osteoclasts was significantly increased in the femoral trabecular bone of OVX mice (Figure 6d). Oral administration of UKT significantly reduced the number of TRAP-positive osteoclasts by OVX (Figure 6d). The osteoclastic index, the extent of the osteoclast surface per bone surface (Oc.S/BS), and the number of osteoclasts per bone surface (N.Oc/BS) were also markedly increased in OVX mice with normal drinking water, whereas the UKT administration significantly decreased Oc.S/BS in OVX mice. The number of osteoclasts per bone volume and the number of osteoclasts per tissue volume also showed the same results (Figure 6e and Figure S2b).

## 3. Discussion

**Figure 7:**
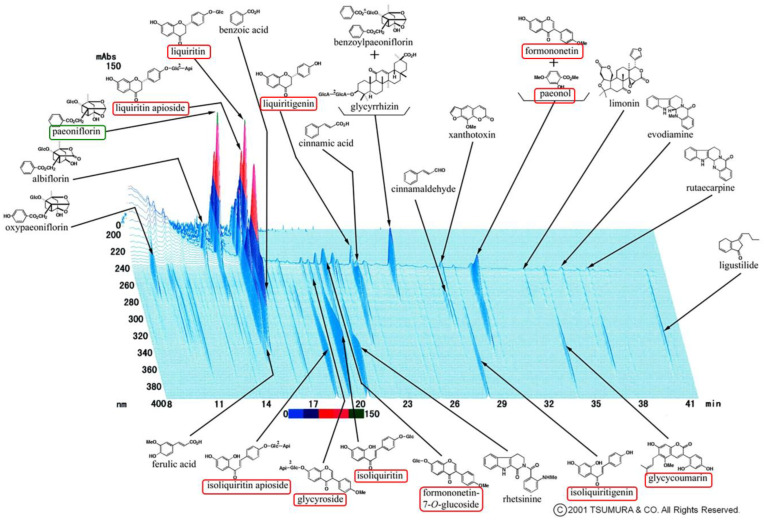
The 3D-HPLC pattern of Unkeito (UKT). The green box shows paeoniflorin, which is a type of terpene glycoside. The red boxes show the active estrogenic components, which are derived from the herb’s or interherb’s new metabolites, i.e., liquiritin apioside, liquiritin, liquirtigenin, formononetin + paeonol, isoliquiritin apioside, glycyroside, isoliquiritin, formononetin-7-O-glucoside, isoliquiritigenin, and glycycoumarin.

Postmenopausal osteoporosis is primarily caused by estrogen deficiency, a critical concern in aging societies. We found 10 chemical components that are all active estrogenic components, which are shown in the red boxes of Figure 7. Previous studies indicated that 17β-estradiol can directly inhibit RANKL-induced osteoclast differentiation and enhance osteoclast apoptosis through caspase-3 activation in RAW 264 cells [17,18]. These reports established that estrogen-protecting effects against postmenopausal osteoporosis are partly mediated through the estrogen receptor. We have previously demonstrated that UKT exhibited strong estrogen receptor beta (ERβ)-dependent estrogenic activity through the cell proliferation bioassay and yeast two-hybrid assay [19]. The herb *Paeonia lactiflora*, which is one of the constituents of UKT, can increase the expression of ERβ in vivo [20]. UKT was also reported to be as effective as 17β-estradiol at preventing OVX-induced bone loss in rats [21]. In this study, we verified that UKT suppressed RANKL-induced osteoclast differentiation and enhanced osteoclast apoptosis in vitro. Moreover, UKT prevented OVX-induced bone loss in a short period of administration in vivo. Both the surface area and number of osteoclasts in bone sections from OVX mice administered with UKT were smaller than those administered with water. Thus, UKT may act on osteoclasts through estrogen-like effects. However, the uterus of UKT-treated OVX mice/sham mice was not altered in terms of either weight gain or enlargement compared to water-treated OVX mice/sham mice. Selective estrogen receptor modulators, which can prevent and treat postmenopausal osteoporosis, can bind the estrogen receptor (ER) and act as ER agonists or antagonists depending on the target tissue [22]. UKT may have this agonistic effect on bone tissue and an antagonistic effect on the uterus, although further investigations are warranted to confirm this.

In the 3D-HPLC analysis of Unkeito (UKT), we found that one of the largest peaks was paeoniflorin (Figure 7 green box). Wang et al. showed that paeoniflorin can suppress osteoclastogenesis and bone resorption by inhibiting the NF-κB signaling pathway in vitro and in vivo [23]. Similarly, UKT contains rutaecarpine, a major alkaloid that can inhibit osteoclast differentiation in bone marrow-derived macrophages in vitro via the NF-κB signaling pathway [24]. Our data showed that UKT suppressed osteoclastogenesis via the NF-κB signaling pathway. UKT may act on osteoclasts not only through estrogen-like effects, but also in a manner similar to that exhibited by other substances, such as paeoniflorin; however, this requires further investigation.

At the molecular level, the homeostasis of bone metabolism is a delicate balance between positive and negative regulation induced by NFATc1 and Bcl6 in osteoclast differentiation, respectively. Based on previous reports, Bcl6 directly binds to NFATc1, negatively regulating its expression. Furthermore, Bcl6 also negatively regulates the cell fusion factor Dc-stamp and functional gene for bone matrix degradation *Ctsk* through direct binding [25]. In other words, activation of Bcl6 expression is required for the inhibition of osteoclast differentiation, multi-nucleation, and functional expression. Our results indicated that the mRNA and protein expression of NFATc1 were decreased by UKT, while those of Bcl6 were increased. Correspondingly, the mRNA and protein expression of Blimp1, the upstream regulator of Bcl6, decreased. Moreover, the gene expression of *Dcstamp* and *Ctsk* was also decreased by UKT. Other research has indicated that Sirt6 expressed by RANKL-induced NFATc1 forms a complex with Blimp1 that negatively regulates the expression of anti-osteoclastogenic genes, such as *Mafb* [26]. However, the mRNA expression levels of *Mafb* did not differ between the UKT and control groups in our study (Figure S3a). These results suggest that UKT not only suppressed osteoclastogenesis but also inhibited multinuclear maturation and the resorptive function of osteoclasts on bone through the Blimp1–Bcl6 axis. 

In addition, the NF-κB signaling pathway is a highly sensitive method for the differentiation of RANK-expressing osteoclast precursors into TRAP-positive osteoclasts in response to RANKL. Therefore, upon stimulation by RANKL, nuclear factor of kappa light polypeptide gene enhancer in B-cells inhibitor, alpha (IκBα), is rapidly phosphorylated by the IκB kinase complex leading to ubiquitination and subsequent degradation of IκBα by the proteasome, followed by nuclear translocation of the p65/p50 heterodimer. When the p65/p50 heterodimer enters the nucleus, it can induce transient auto-amplification of NFATc1 expression [6,7,27]. The mRNA and protein expression levels of IκBα did not differ between the UKT and control groups (data not shown). The nucleus translocation factor p65 could directly and positively regulate NFATc1, as described previously [6,7]. In this study, both mRNA and protein expression levels of p65 were decreased by UKT, and p65 expression was also reduced in the nucleus. This leads to the inhibition of the downstream promoter NFATc1, which directly affects the expression of genes during osteoclast differentiation, such as *Trap*, *Mmp9*, and *Ctsk*, all of which show reduced expression with UKT. Further, the MAPK signaling pathway is also an important pathway in response to RANKL-RANK, classified into ERK, p38, and JNK based on the homology of primary sequences, and c-Fos is the transcription factor downstream of MAPK [8,9,10]. We therefore examined the mRNA expression levels of ERK, p38, JNK, and c-Fos and found no significant differences following treatment with UKT (Figure S3b). This is, to the best of our knowledge, the first validation of the effectiveness of UKT to inhibit osteoclast differentiation through both negative and positive signaling pathways.

UKT was the most effective at preventing not only the differentiation of precursor osteoclasts into mononuclear osteoclasts during the early stage, but also in preventing fusion into mature osteoclasts during the late stage of differentiation. Thus, apoptosis of osteoclasts is regulated by caspase-3 [28]. The cleaved form of caspase-3 is responsible for much of the proteolysis during apoptosis; hence, its detection is considered a reliable marker for cells that are dying or have died [29]. Our data found that UKT enhanced RANKL-induced apoptosis of mononuclear osteoclasts by the activation of caspase-3, which may play an inhibitory role at the late stage of RANKL-induced osteoclast differentiation. This may be another reason for the inhibition of osteoclast maturation. However, we found that mature osteoclasts did not undergo apoptosis following treatment with UKT for 24 h (data not shown). UKT may have an apoptosis-inducing effect only on mononuclear osteoclasts formed during early differentiation, but not on mature and functional osteoclasts. In recent studies, osteoclast-derived apoptotic bodies played a bridging role in osteoclast–osteoblast coupling in bone remodeling [30]. In osteoclastogenesis, osteoclast-derived exosomes were transferred to osteoblasts, inhibiting bone formation [31,32]. Therefore, osteoclast differentiation inhibition and apoptosis not only affect bone resorption, but also have a direct impact on osteoblasts and bone formation. To further elucidate the overall role of UKT in the dynamic balance of bone metabolism, the effects and mechanisms of UKT on osteoblasts warrant further investigation.

Based on the characteristics of UKT in the clinical treatment of menopausal symptoms, we screened KSS, KKT, and NYT. These Kampo medicines suppressed osteoclast differentiation without cytotoxicity. In particular, UKT and KSS effectively inhibited osteoclast differentiation from the early stage and further inhibited the formation of mature osteoclasts in the mid-differentiation stage, although the effect of KSS was slightly weaker than that of UKT. However, KKT and NYT were effective in the early stage; the formation of mature osteoclasts in the middle stage was slightly affected. We described above that paeoniflorin, which is a major component involved in UKT, may inhibit osteoclastogenesis. Paeoniflorin was also involved in KSS and NYT, but few in KTT. The peak of paeoniflorin in UKT was higher than KSS, meaning that the amount of paeoniflorin in UKT was much more than in KSS. On the other hand, the amount of paeoniflorin in NYT was lower than both UKT and KSS (Figure 7 and Figure S4). It is possible that this is one of the reasons that UKT is more effective for the inhibitory activity in osteoclastogenesis than the other three. In addition, liquiritin and liquiritin apioside were common estrogenic components involved in these four, and the amount of them was higher than the other estrogenic components in these four from 3D-HPLC data (Figure 7 and Figure S4). To date there has been no study about the effects of liquiritin and liquiritin apioside on osteoclastogenesis. We hypothesize that liquiritin and liquiritin apioside may have a role in inhibiting osteoclasts for the protection of osteoporosis, which is potentially a new subject to be explored.

**Figure 8:**
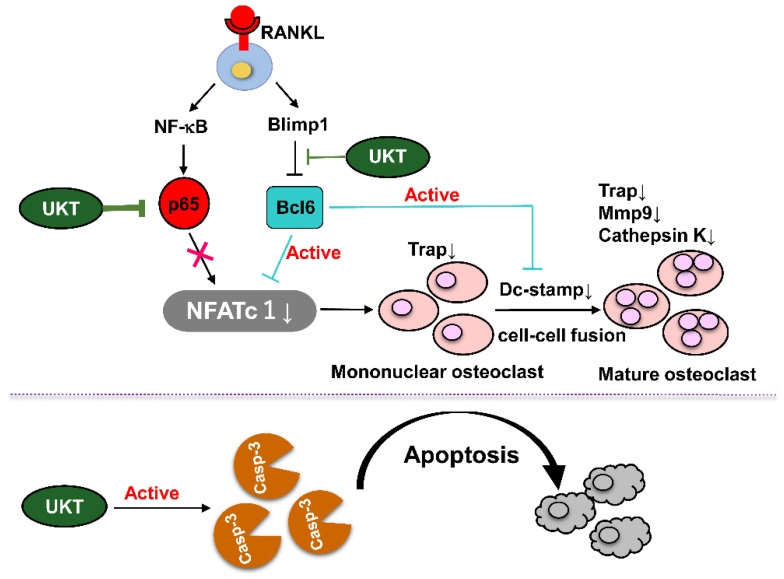
Unkeito (UKT) suppresses RANKL-mediated osteoclastogenesis through the NF-κB signaling pathway and the Blimp1–Bcl6 axis and induces mononuclear osteoclast apoptosis via caspase-3 activation. Thus, UKT may serve as a potential therapeutic agent for osteoporosis. Arrows for activation, lines for inhibition.

We found that UKT can suppress osteoclast differentiation and induces apoptosis of mononuclear osteoclasts (Figure 8). However, the active ingredients from UKT extracts that block the NF-κB signaling pathway and Blimp1–Bcl6 axis have not been identified. We would like to analyze and screen the active ingredients of UKT in the future to investigate the possible direct mechanisms.

## 4. Materials and Methods

### 4.1. Reagents

Recombinant human soluble RANKL (sRANKL) was purchased from Oriental Yeast Co., Ltd. (Tokyo, Japan).

### 4.2. Extraction of Active Ingredients from Kampo Medicines

The extract powders of UKT (TJ-106), KSS (TJ-24), KKT (TJ-137), and NYT (TJ-108) were purchased from Tsumura & Co. (Tokyo, Japan). The extract powders of Kampo medicines were prepared by hot water extraction (decoction) of the crude drugs, which are listed in Table S1. Each of the extracted powders was mixed 1:10 with 99.5% ethanol, and the supernatant was filter sterilized using 0.45-µm membrane filters (Toyo Roshi Kaisha, Ltd., Tokyo, Japan). The liquid was dehydrated in a vacuum and then resuspended using dimethyl sulfoxide (DMSO; Nacalai Tesque. Inc., Kyoto, Japan) to completely dissolve the concentrate.

### 4.3. Osteoclast Differentiation

The mouse leukemic monocyte–macrophage cell line RAW 264 was purchased from the Japan Riken BioResource Research Center (RCB0535) (Ibaraki, Japan). RAW264 cells (1 × 10^3^ cells/well) were seeded into 96-well plates and cultured in α-minimal essential medium (Nacalai Tesque Inc., Kyoto, Japan) containing 10% fetal bovine serum (HyClone, Logan, UT, USA) and 100 units/mL penicillin–streptomycin (Thermo Fisher Scientific, Waltham, MA, USA). Kampo medicines at the concentration of 300 µg/mL in 0.1% DMSO or 0.1% DMSO (control) were added to the wells. Cells were incubated at 37 °C in a 5% CO_2_ atmosphere for three to five days with 100 ng/mL sRANKL to induce osteoclast formation. Osteoclasts were confirmed by TRAP staining. TRAP-positive mononuclear (nuclei/cell = 1) and multinucleated osteoclasts (nuclei/cell ≥ 3) were counted under a microscope.

### 4.4. TRAP Activity Assay

The TRAP activity assay was performed using 10 mM pNPP (p-nitrophenyl phosphate, disodium salt) (Nacalai Tesque Inc.), which was mixed with tartrate-containing buffer with 0.2 M sodium chloride (Sigma-Aldrich, St. Louis, MO, USA), 1 mM L(+)-ascorbic acid (FUJIFILM Wako Pure Chemical Corporation, Tokyo, Japan), 10 mM sodium(+)-tartrate dehydrate (Nacalai Tesque Inc.), and 0.1% Triton X-100 (Sigma-Aldrich), which was further mixed with 0.1 M sodium acetate (Tokyo Chemical Industry Co., Ltd., Tokyo, Japan) buffer (pH 5.2). The supernatant of the cell culture was mixed with the same volume of tartrate-containing buffer and incubated at 37 °C for 1 h. The reaction was stopped using 0.3 N sodium hydroxide solution and measured at OD_405 nm_ using a microplate reader (Bio-Rad Laboratories, Hercules, CA, USA).

### 4.5. TRAP Staining

TRAP staining was performed using 0.23 mM naphthol AS-MX phosphate (Nacalai Tesque Inc.) and 1.3 mM fast red violet LB salt (Sigma-Aldrich), which were dissolved by *N*,*N*-dimethylformamide (FUJIFILM Wako Pure Chemical Corporation), and then mixed with 50 mM tartrate-containing buffer (pH 5.0). The tartrate-containing buffer was composed of sodium acetate, 50 mM sodium tartrate dehydrate (Nacalai Tesque Inc.), and 1% acetic acid (FUJIFILM Wako Pure Chemical Corporation). Samples were fixed with 4% paraformaldehyde on ice for 10 min, stained with TRAP staining reagent, and incubated at 37 °C for 1 h. The TRAP-stained cells were observed using a microscope (Nikon ECLIPSE Ts2). Image analysis was performed with NIS-Elements Analysis Software (Nikon Solutions Co., Ltd., Tokyo, Japan). The numbers of TRAP-positive mononuclear osteoclasts (nuclei/cell = 1) and mature osteoclasts (nuclei/cell ≥ 3 or 10) were counted and calculated by averaging the two 96-well field of views from three independent experiments.

### 4.6. Quantitative Real-Time PCR Analysis of Gene Expression

To evaluate the expression of osteoclast markers, RAW 264 cells were seeded at 1 × 10^3^ per well in a 96-well plate and induced using 100 ng/mL sRANKL with or without Kampo medicine to differentiate into osteoclasts for one, three, or five days. qPCR analysis was performed using the CellAmp™ Direct TB Green^®^ RT-qPCR kit (TaKaRa, Kusatsu, Japan) and Rotor-Gene Q real-time PCR system (QIAGEN, Dusseldorf, Germany) according to the manufacturer’s instructions. To verify the specificity of each primer, a melting-curve analysis was performed by fluorescence every 0.5 °C from 60 °C to 95 °C. The absence of contamination from either genomic DNA amplification or primer dimer formation was ensured with a Milli-Q water template control. Gene expression values of markers were normalized to *Gapdh.* The sequences of the gene-specific primer pairs can be found in Table S2.

### 4.7. Immunocytochemistry Staining

RAW 264 cells (1 × 10^3^) were seeded into an 8-well chamber slide (Thermo Fisher Scientific), stimulated by RANKL to differentiate into osteoclasts, and incubated with UKT. After culturing for three days, the cells were fixed with 4% paraformaldehyde-phosphate buffer solution for 20 min, permeabilized with 0.3% Triton X-100 for 20 min, and blocked with Blocking One for 1 h. Chamber slides were incubated with rabbit anti-NFATc1 (1:100; D15F1, Cell Signaling Technology, Inc., Danvers, MA, USA), rabbit anti-NF-κB p65 (1:100; Poly6226, BioLegend, Inc., San Diego, CA, USA), or Blimp1 (1:100; C14A4, Cell Signaling Technology, Inc.) overnight at 4 °C, followed by incubation with secondary antibody Cy3 conjugated donkey anti-rabbit-IgG (1:200; BioLegend, Inc.) or Alexa Fluor 488-conjugated anti-rabbit-IgG (1:500, Jackson ImmunoResearch, Baltimore, MD, USA), and Texas red conjugated anti-mouse-IgG (1:200) for 1 h. All the secondary antibodies were confirmed to be undetected in the absence of primer antibodies, as shown in Figure S5. Finally, cells were stained with 4′,6-diamidino-2-phenylindole (DAPI) (Dojindo, Kumamoto, Japan) for nuclear staining and observed by confocal laser scanning microscopy using an FV3000 system (Olympus, Tokyo, Japan). The images were analyzed using cellSens Dimension software (Olympus).

### 4.8. Apoptosis Assay

RAW 264 cells (1 × 10^3^) were seeded into 96-well plates and stimulated with 125 ng/mL sRANKL to differentiate into osteoclasts. After culturing for three days, the cells were treated with 100 and 300 µg/mL UKT for 24 h. The cell lysates were analyzed using qPCR. RAW 264 cells (1 × 10^3^) were then seeded into 8-well chamber slides, stimulated by 125 ng/mL sRANKL for three days, and treated with 100 µg/mL UKT for 6 h to induce osteoclast apoptosis. Immunocytochemical staining was performed with anti-cleaved caspase-3 (1:200; Cell Signaling Technology, Inc.) and anti-TRAP (1:100; Santa Cruz Biotechnology Inc., Dallas, TX, USA) antibodies overnight at 4 °C, followed by incubation with secondary antibody Alexa Fluor 488-conjugated anti-rabbit-IgG (1:500, Jackson ImmunoResearch Inc., West Grove, PA, USA) and Texas red conjugated anti-mouse-IgG (1:200) for 1 h at room temperature (24 °C). Cells nuclei were stained with DAPI and observed by confocal laser scanning microscopy using an FV3000 system. Images were analyzed using cellSens Dimension software.

### 4.9. Animals

Eight-week-old specific pathogen-free (SPF) grade female CD-1 mice were obtained from Japan SLC, Inc. (Hamamatsu, Japan). Forty-four mice were divided into 4 experimental groups: (1) sham + normal drinking water, (2) sham + 3% UKT, (3) OVX + normal drinking water, and (4) OVX + 3% UKT. They were subjected to bilateral ovariectomy surgery (OVX) under anesthesia with 2% isoflurane (FUJIFILM Wako Pure Chemical Corporation), and maintained using 0.5–1% isoflurane. Immediately after the surgery, the mice were given daily oral supplementation of normal diet with tap water or 3% UKT in drinking water and weighed every week. Food intake and drinking volume were checked every 3 days during the experiment period. 

On day 28 post-surgery, the mice were euthanized under anesthesia for collection of blood and tissues. The animal experiments were carried out in strict accordance with the guidelines of the Japanese Society for Pharmacology and were approved by the Committee of Animal Care at Kansai Medical University (approval number: #21-042).

### 4.10. Bone Analysis

At the end of the experiments, the mice were sacrificed under anesthesia. Their uteruses were removed and weighed. Both femurs were fixed with 4% paraformaldehyde phosphate buffer solution for bone histomorphometric analyses. The right side of the distal femoral trabecular bone was analyzed using micro-computed tomography (SkyScan 1174; SkyScan, Aartselaar, Belgium) for 3D image reconstruction. Images were acquired using the following settings: 50 kV voltage, 770 mA current, and 6.5 µm voxel resolution. Analysis of bone volume per tissue volume ratio (BV/TV), trabecular number (Tb.N), Tb.Sp, and trabecular thickness (Tb.Th) was made at an area within 1 mm from 0.8 mm under the growth plate peak. The left side of the digital femoral embedded block was sliced at 5 µm thickness. Osteoclast parameters were analyzed via staining with aniline blue and TRAP. Analyses were performed with HistoMorph open source software developed by the Institute of Ageing and Chronic Disease, University of Liverpool [33].

### 4.11. Statistical Analysis

Statistical analysis was performed using GraphPad Prism version 8.4.2. All data were presented as mean ± standard error of the mean. For comparisons of in vitro experiments, data were analyzed using Tukey’s multiple comparisons tests. For comparisons of in vivo experiments, two-way analysis of variance was used, followed by the Tukey’s multiple comparison tests. Statistical significance was set at *p* < 0.05.

## 5. Conclusions

We demonstrated that UKT, KSS, KKT, and NYT suppressed RANKL-induced osteoclast differentiation at different stages in RAW 264 cells. For the first time, our results demonstrated that UKT could suppress osteoclast differentiation via the RANKL-mediated Blimp1–Bcl6 axis negative pathway and NF-κB-positive pathway, while enhancing mononuclear osteoclast apoptosis in vitro. Moreover, in this study, the preventive effect of UKT on bone loss in vivo was also determined. Thus, UKT may serve as a potential therapeutic agent for postmenopausal osteoporosis, together with the treatment of menopausal syndromes.

## Data Availability

The data presented in this study are available on request from the corresponding author. The data are not publicly available due to privacy.

## Supplementary Materials

The following supporting information can be downloaded at: https://www.mdpi.com/article/10.3390/ijms23147814/s1.

## References

[1] Johnell O., Kanis J.A. (2006). An estimate of the worldwide prevalence and disability associated with osteoporotic fractures. Osteoporos. Int..

[2] Siris E.S., Brenneman S.K., Barrett-Connor E., Miller P.D., Sajjan S., Berger M.L., Chen Y.T. (2006). The effect of age and bone mineral density on the absolute, excess, and relative risk of fracture in postmenopausal women aged 50-99: Results from the National Osteoporosis Risk Assessment (NORA). Osteoporos. Int..

[3] Inui T., Ishibashi O., Origane Y., Fujimori K., Kokubo T., Nakajima M. (1999). Matrix metalloproteinases and lysosomal cysteine proteases in osteoclasts contribute to bone resorption through distinct modes of action. Biochem. Biophys. Res. Commun..

[4] Saitoh Y., Koizumi K., Sakurai H., Minami T., Saiki I. (2007). RANKL-induced down-regulation of CX3CR1 via PI3K/Akt signaling pathway suppresses fractalkine/CX3CL1-induced cellular responses in RAW264.7 cells. Biochem. Biophys. Res. Commun..

[5] Lam J., Takeshita S., Barker J.E., Kanagawa O., Ross F.P., Teitelbaum S.L. (2000). TNF-alpha induces osteoclastogenesis by direct stimulation of macrophages exposed to permissive levels of RANK ligand. J. Clin. Investig..

[6] Boyce B.F., Xiu Y., Li J., Xing L., Yao Z. (2015). NF-κB-mediated regulation of osteoclastogenesis. Endocrinol. Metab..

[7] Soysa N.S., Alles N. (2009). NF-kappaB functions in osteoclasts. Biochem. Biophys. Res. Commun..

[8] Li X., Udagawa N., Itoh K., Suda K., Murase Y., Nishihara T., Suda T., Takahashi N. (2002). p38 MAPK-mediated signals are required for inducing osteoclast differentiation but not for osteoclast function. Endocrinology.

[9] Chang E.J., Ha J., Huang H., Kim H.J., Woo J.H., Lee Y., Lee Z.H., Kim J.H., Kim H.H. (2008). The JNK-dependent CaMK pathway restrains the reversion of committed cells during osteoclast differentiation. J. Cell Sci..

[10] Kim H.H., Chung W.J., Lee S.W., Chung P.J., You J.W., Kwon H.J., Tanaka S., Lee Z.H. (2003). Association of sustained ERK activity with integrin beta3 induction during receptor activator of nuclear factor kappaB ligand (RANKL)-directed osteoclast differentiation. Exp. Cell Res..

[11] Takayanagi H., Kim S., Koga T., Nishina H., Isshiki M., Yoshida H., Saiura A., Isobe M., Yokochi T., Inoue J. (2002). Induction and activation of the transcription factor NFATc1 (NFAT2) integrate RANKL signaling in terminal differentiation of osteoclasts. Dev. Cell.

[12] Nomiyama H., Egami K., Wada N., Tou K., Horiuchi M., Matsusaki H., Miura R., Yoshie O., Kukita T. (2005). Identification of genes differentially expressed in osteoclast-like cells. J. Interferon Cytokine Res..

[13] Amarasekara D.S., Yun H., Kim S., Lee N., Kim H., Rho J. (2018). Regulation of osteoclast differentiation by cytokine networks. Immune Netw..

[14] Miyauchi Y., Ninomiya K., Miyamoto H., Sakamoto A., Iwasaki R., Hoshi H., Miyamoto K., Hao W., Yoshida S., Morioka H. (2010). The Blimp1-Bcl6 axis is critical to regulate osteoclast differentiation and bone homeostasis. J. Exp. Med..

[15] Kuo Y.J., Tsuang F.Y., Sun J.S., Lin C.H., Chen C.H., Li J.Y., Huang Y.C., Chen W.Y., Yeh C.B., Shyu J.F. (2012). Calcitonin inhibits SDCP-induced osteoclast apoptosis and increases its efficacy in a rat model of osteoporosis. PLoS ONE.

[16] Nair A.B., Jacob S. (2016). A simple practice guide for dose conversion between animals and human. J. Basic Clin. Pharm..

[17] Katao Y., Sawai H., Inami K., Domae N., Matsumoto N. (2011). Direct effects of estrogen on differentiation and apoptosis of osteoclasts. Orthod. Waves.

[18] Kameda T., Mano H., Yuasa T., Mori Y., Miyazawa K., Shiokawa M., Nakamaru Y., Hiroi E., Hiura K., Kameda A. (1997). Estrogen inhibits bone resorption by directly inducing apoptosis of the bone-resorbing osteoclasts. J. Exp. Med..

[19] Wang Z., Kanda S., Shimono T., Enkh-Undraa D., Nishiyama T. (2018). The *in vitro* estrogenic activity of the crude drugs found in Japanese herbal medicines prescribed for menopausal syndrome was enhanced by combining them. BMC Complement. Altern. Med..

[20] Wang J., Song C., Gao D., Wei S., Sun W., Guo Y., Sun S., Tian X., Li H., Qiao M. (2020). Effects of *Paeonia lactiflora* extract on estrogen receptor *β*, TPH2, and SERT in rats with PMS anxiety. BioMed Res. Int..

[21] Hidaka S., Okamoto Y., Nakajima K., Suekawa M., Liu S.Y. (1997). Preventive effects of traditional Chinese (Kampo) medicines on experimental osteoporosis induced by ovariectomy in rats. Calcif. Tissue Int..

[22] Kulak J., Kulak C.A., Taylor H.S. (2010). SERMs in the prevention and treatment of postmenopausal osteoporosis: An update. Arq. Bras. Endocrinol. Metabol..

[23] Wang Y., Dai J., Zhu Y., Zhong W., Lu S., Chen H., Chai Y. (2018). Paeoniflorin regulates osteoclastogenesis and osteoblastogenesis via manipulating NF-κB signaling pathway both in vitro and in vivo. Oncotarget.

[24] Fukuma Y., Sakai E., Komaki S., Nishishita K., Okamoto K., Tsukuba T. (2018). Rutaecarpine attenuates osteoclastogenesis by impairing macrophage colony stimulating factor and receptor activator of nuclear factor κ-B ligand-stimulated signalling pathways. Clin. Exp. Pharmacol. Physiol..

[25] Nishikawa K., Nakashima T., Hayashi M., Fukunaga T., Kato S., Kodama T., Takahashi S., Calame K., Takayanagi H. (2010). Blimp1-mediated repression of negative regulators is required for osteoclast differentiation. Proc. Natl. Acad. Sci. USA.

[26] Park S.J., Huh J.E., Shin J., Park D.R., Ko R., Jin G.R., Seo D.H., Kim H.S., Shin H.I., Oh G.T. (2016). Sirt6 cooperates with Blimp1 to positively regulate osteoclast differentiation. Sci. Rep..

[27] Ghosh S., May M.J., Kopp E.B. (1998). NF-kappa B and Rel proteins: Evolutionarily conserved mediators of immune responses. Annu. Rev. Immunol..

[28] Tanaka S., Wakeyama H., Akiyama T., Takahashi K., Amano H., Nakayama K.I., Nakamura K. (2010). Regulation of osteoclast apoptosis by Bcl-2 family protein Bim and Caspase-3. Adv. Exp. Med. Biol..

[29] Crowley L.C., Waterhouse N.J. (2016). Detecting cleaved caspase-3 in apoptotic cells by flow cytometry. Cold Spring Harb. Protoc..

[30] Ma Q., Liang M., Limjunyawong N., Dan Y., Xing J., Li J., Xu J., Dou C. (2020). Osteoclast-derived apoptotic bodies show extended biological effects of parental cell in promoting bone defect healing. Theranostics.

[31] Sun W., Zhao C., Li Y., Wang L., Nie G., Peng J., Wang A., Zhang P., Tian W., Li Q. (2016). Osteoclast-derived microRNA-containing exosomes selectively inhibit osteoblast activity. Cell Discov..

[32] Li D., Liu J., Guo B., Liang C., Dang L., Lu C., He X., Cheung H.Y., Xu L., Lu C. (2016). Osteoclast-derived exosomal miR-214-3p inhibits osteoblastic bone formation. Nat. Commun..

[33] Van’t Hof R.J., Rose L., Bassonga E., Daroszewska A. (2017). Open source software for semi-automated histomorphometry of bone resorption and formation parameters. Bone.

