# Supplementary material for: Unkeito Suppresses RANKL-Mediated Osteoclastogenesis via the Blimp1–Bcl6 and NF-κB Signaling Pathways and Enhancing Osteoclast Apoptosis
Source: Int J Mol Sci. 2022 Jul 15;23(14):7814. doi: 10.3390/ijms23147814 (PMC9323376; doi:10.3390/ijms23147814)
Supplement: Supplementary file 1 [file ijms-23-07814-s001.zip › ijms-1788743-supplementary.pdf]

Supplementary data

Unkeito Suppresses RANKL-Mediated Osteoclastogenesis *Via* the Blimp1-Bcl6 and NF-κB Signaling Pathways and Enhancing Osteoclast Apoptosis

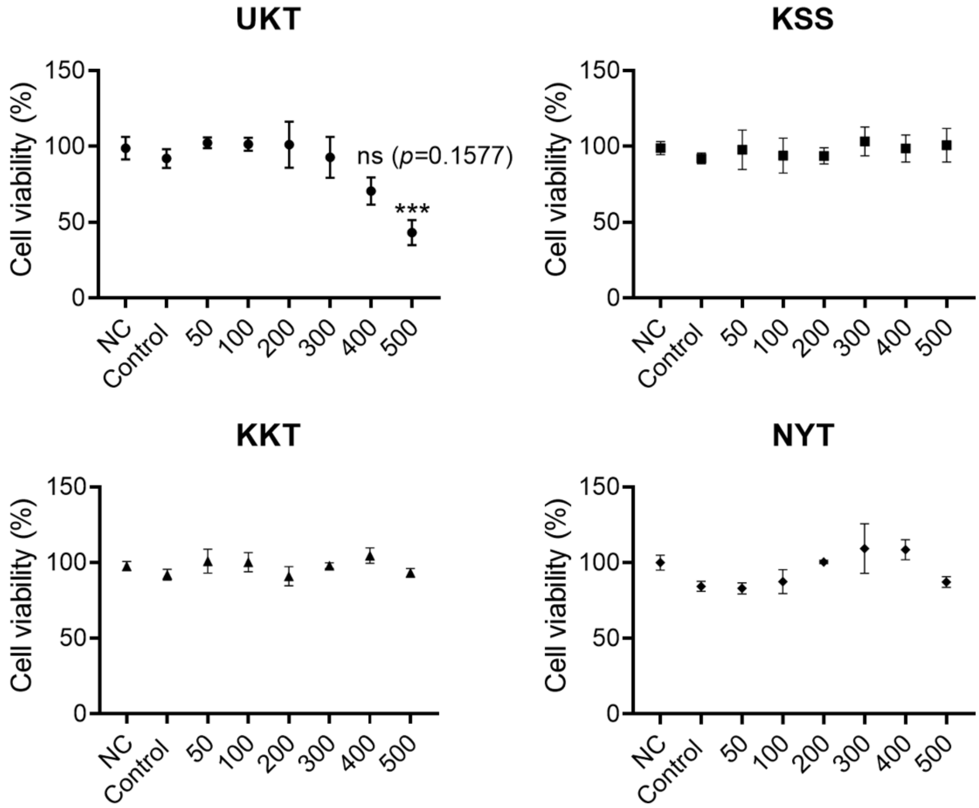

Figure S1. Cell viability of Unkeito (UKT), Kamishoyosan (KSS), Kamikihito (KKT), and Ninjinyoeito (NYT) in RAW 264 cells. RAW 264 cells were seeded at 2×10^4 per well in 96-well plates, cultured overnight, and treated with various concentrations of Kampo medicine extracts at 37 °C in a 5% CO_2 atmosphere for 24 h. Next, 10 μL of CCK-8 solution was added to each well and incubated at 37 °C for 1 h. The absorbance (optical density, OD 450 nm) was measured (n = 4). Error bars represent ± standard error of the mean. ns, not significant differences, *** $p < 0.005$ versus the control groups; NC: negative control

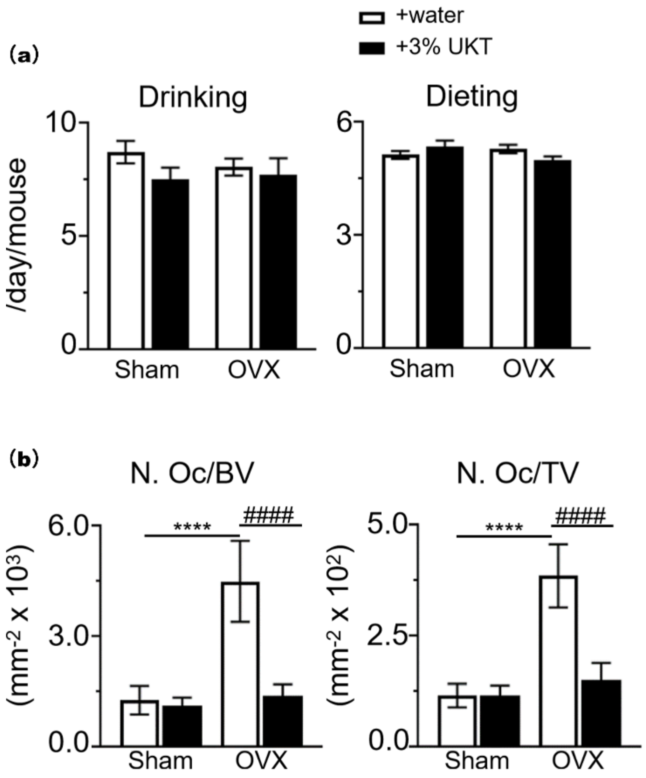

Figure S2. Eight-week aged CD-1 mice were daily administrated Unkeito (UKT) in drinking water for 28 consecutive days, following sham or ovariectomy (OVX)-operation (sham + water, n = 11; sham + 3% UKT, n = 11; OVX + water, n = 11; OVX + 3% UKT, n = 11). Open bar represents mice with water, while closed bar represents mice treated with 3% UKT. (**a**) Average of drinking volume and food intake/day/mouse; (**b**) Quantitative data from histomorphometric analysis. Two-way ANOVA was used, followed by the Tukey's multiple comparison tests. Data expressed as means ± standard error of mean for each group. ****$p < 0.0001$, vs. sham-operated mice with water. ####$p < 0.0001$, vs. OVX-operated mice with water. N.Oc/BV, osteoclast number/bone volume; N.Oc/TV, osteoclast number/tissue volume

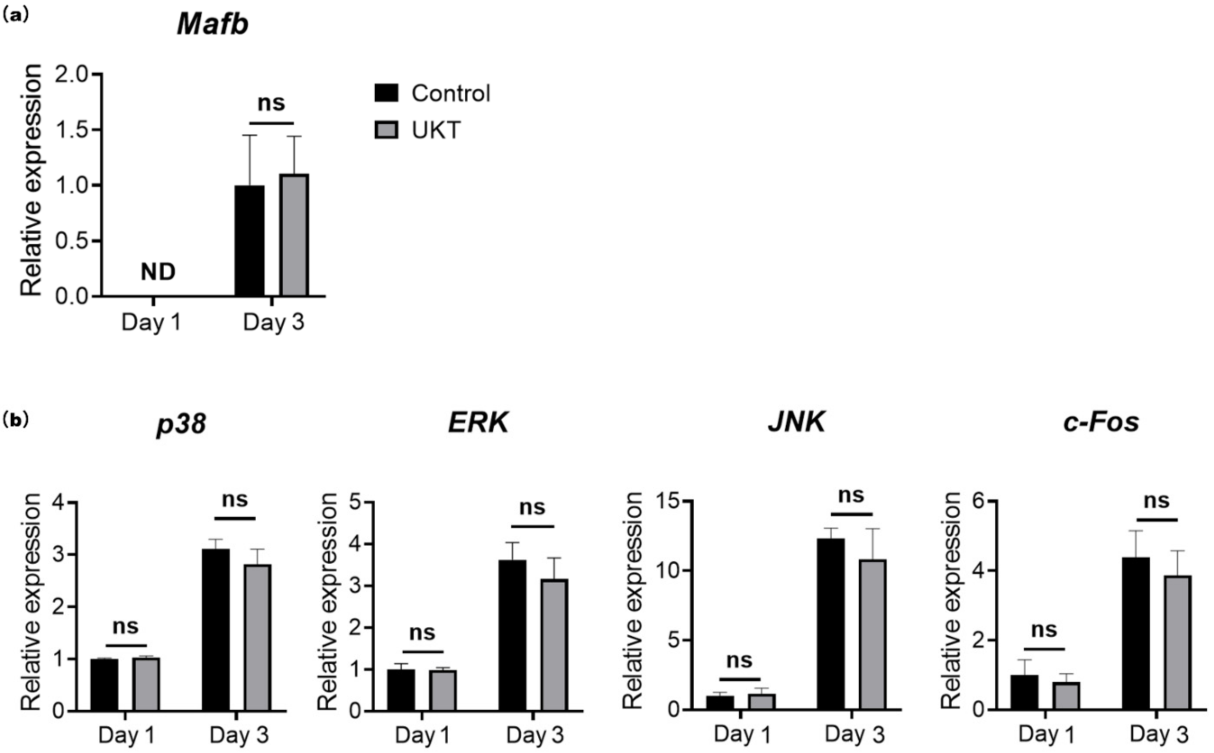

Figure S3. Unkeito (UKT) has no effect on the inhibition of the MAPK pathway and Blimp1-Mafb axis. The mRNA expression levels of (**a**) *Mafb*, (**b**) *p38, ERK, JNK,* and *c-Fos* normalized to that of *Gapdh* (n = 3). Error bars represent ± standard error of mean. ns, not significant versus the control groups (black box); ND: not detected

(a)

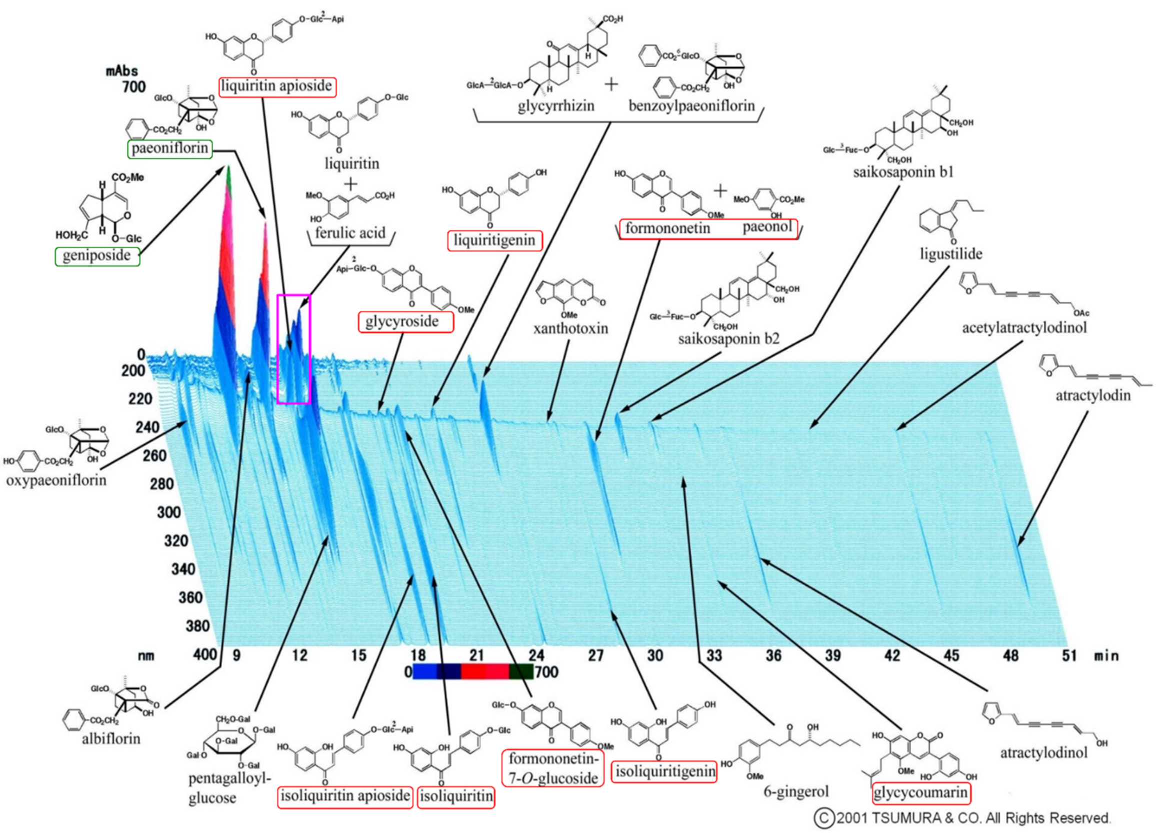
mAbs
700
liquiritin apioside
paeoniflorin
geniposide
liquiritin
ferulic acid
liquiritigenin
glycyroside
glycyrrhizin
benzoylpaeoniflorin
formononetin
paeonol
xanthotoxin
saikosaponin b2
saikosaponin b1
ligustilide
acetylatractylodinol
atractylodin
oxypaeoniflorin
albiflorin
pentagalloyl-glucose
isoliquiritin apioside
isoliquiritin
formononetin-7-O-glucoside
isoliquiritigenin
6-gingerol
glycycoumarin
atractylodinol
nm
min
©2001 TSUMURA & CO. All Rights Reserved.

\

(b)

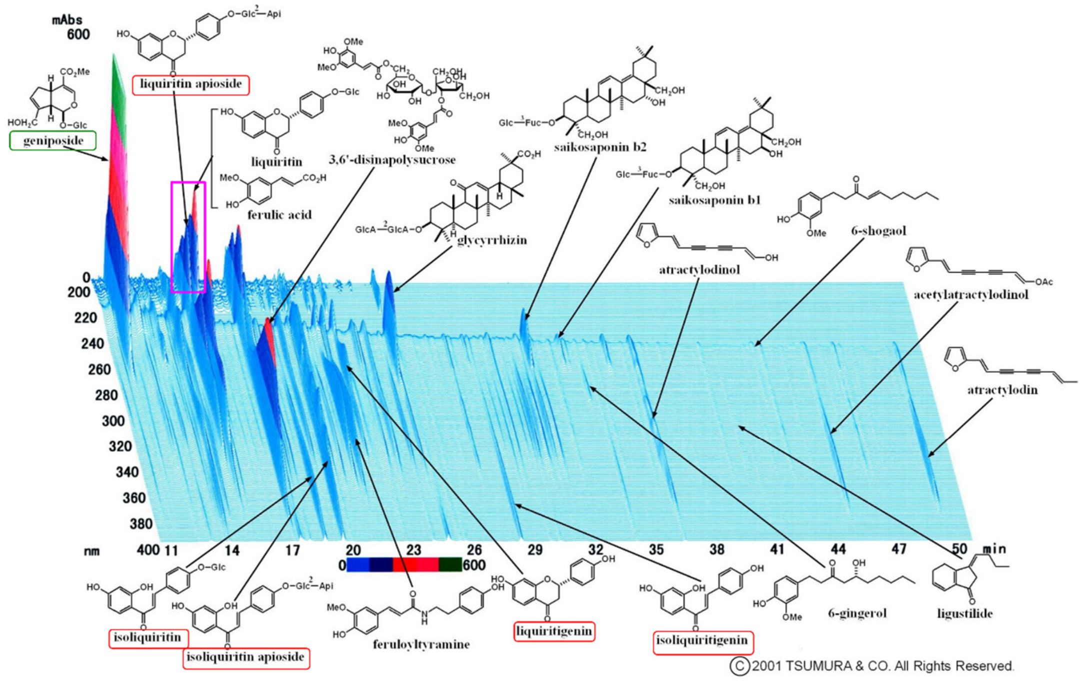
mAbs
600
liquiritin apioside
geniposide
liquiritin
ferulic acid
3,6'-disinapolysucrose
glycyrrhizin
saikosaponin b2
saikosaponin b1
atractylodinol
6-shogaol
acetylatractylodinol
atractylodin
isoliquiritin
isoliquiritin apioside
feruloyltyramine
liquiritigenin
isoliquiritigenin
6-gingerol
ligustilide
nm
min
©2001 TSUMURA & CO. All Rights Reserved.

(c)

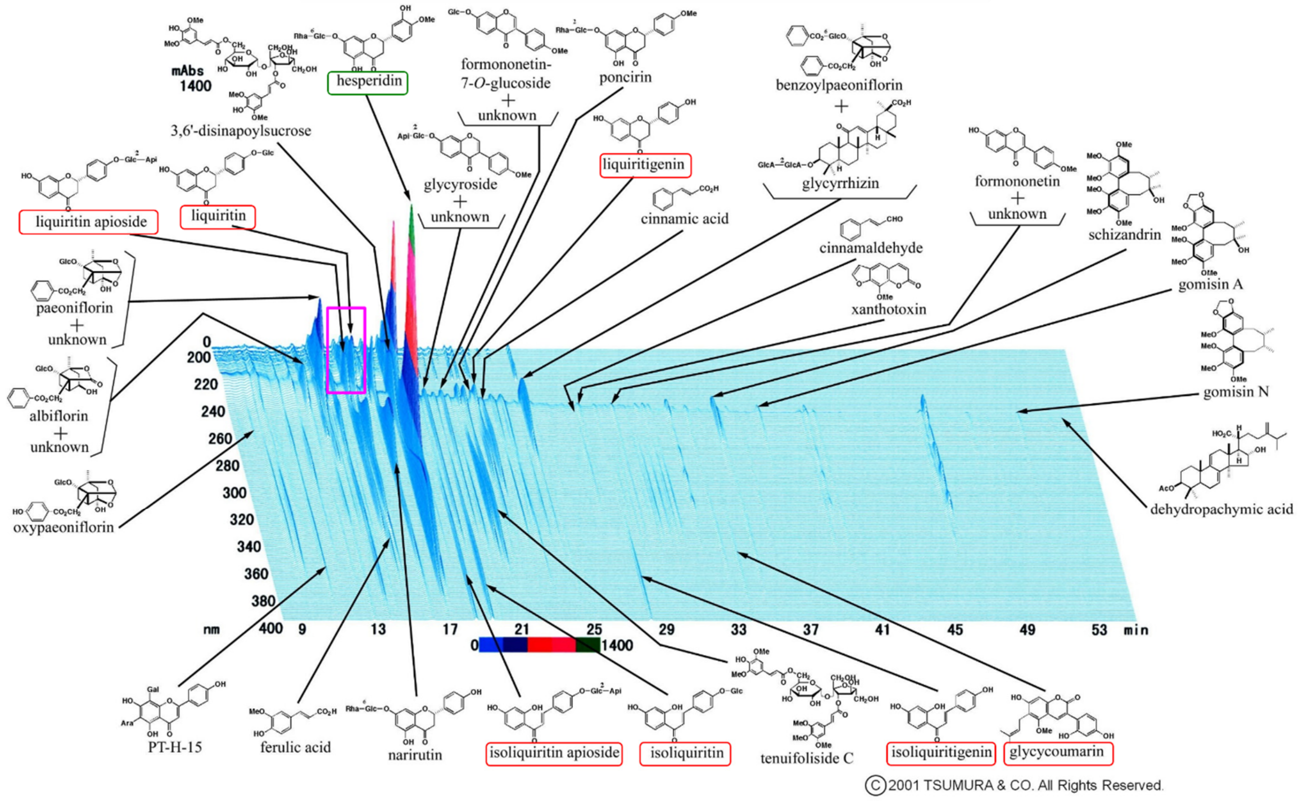

Figure S4. 3D-HPLC pattern of Kamishoyosan (a), Kamikihito (b), Ninjinyoeito (c). The green boxes is paeoniflorin,. The red boxes show the active estrogenic components, which are derived from the herb's or interherb's new metabolites, i.e. Liquiritin apioside, Liquiritin, Liquirtigenin, Formononetin+Paeonol, Isoliquiritin apioside, Glycyroside, Isoliquiritin, Formononet-in-7-O-glucoside, Isoliquiritigenin, Glycycoumarin. The pink box is the peak of Liquiritin apioside and Liquiritin.

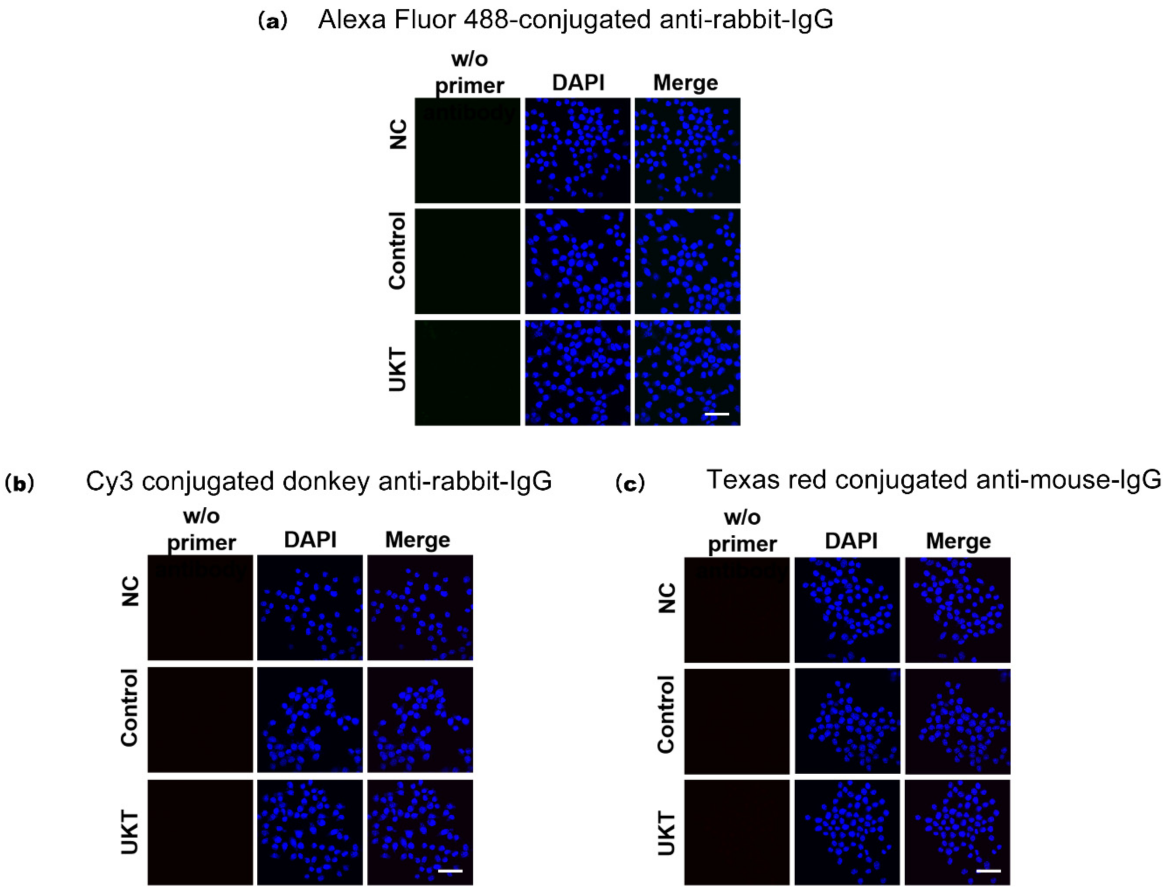

Figure S5. Secondary antibodies used for immunocytochemistry staining without primer antibodies were not detected. (**a**) Alexa Fluor 488-conjugated anti-rabbit-IgG (1:500); (**b**) Cy3 conjugated donkey anti-rabbit-IgG (1:200); (**c**) Texas red conjugated anti-mouse-IgG (1:200). Cells were stained with 4′,6-diamidino-2-phenylindole (DAPI) for nuclear staining and observed using confocal laser scanning microscopy using an FV3000 system (Olympus). Images were analyzed using the cellSens Dimension software (Olympus) (scale bar = 10 μm)

Table S1. Kampo medicine components

Name			
**Unkeito UKT (TJ-106)**	**Kamishoyosan KSS (TJ-24)**	**Kamikihito KKT (TJ-137)**	**Ninjinyoeito NYT (TJ-108)**
**Component**			
Pinellia tuber	Japanese angelica root	Ginseng	Ginseng
Ophiopogon tuber	Peony root	Poria sclerotium	Japanese angelica root
Japanese angelica root	Poria sclerotium	Bupleurum root	Poria sclerotium
Peony root	Bupleurum root	Jujube seed	Rehmannia root
Moutan bark	Moutan bark	Longan arillus	Cinnamon bark
Ginseng	Gardenia fruit	Astragalus root	Citrus unshiu peel
Glycyrrhiza	Glycyrrhiza	Japanese angelica root	Peony root
Cnidium rhizome	Peppermint	Gardenia fruit	Polygala root
Cinnamon bark	Ginger	Polygala root	Schisandra fruit
Evodia fruit		Jujube	Glycyrrhiza
Ginger		Glycyrrhiza	Astragalus root
		Saussurea root	
		Ginger	

KEGG DRUG Database

Table S2. RT-qPCR primer pair sequences and gene probes for cell markers

No.	Gene Name	Forward Primer (5′-3′)	Reverse Primer (5′-3′)
1	Mouse *Gapdh*	TGA CTC CAC TCA CGG CAA AT	GGT CTC GCT CCT GGA AGA TG
2	Mouse *Rela (p65) V1*	CCC AGA CCG CAG TAT CCA T	GCT CCA GGT CTC GCT TCT T
3	Mouse *Nf-kb1(p105/50)*	TCT GGG GAT ACT GAA CAA TGC	CCA GAG ACC TCA TAG TTG TCC A
4	Mouse *Bcl6 V2*	CCCACCCTAGGGAGTTGTG	CCCCCTCTCAAAAGATTCACT
5	Mouse *Prdm1 (Blimp1)*	ACGTGTGGGTACGACCTTG	CCATGTCCATTTTCATGATCC
6	Mouse *Nfatc1*	TCC AAA GTC ATT TTC GTG GA	CTT TGC TTC CAT CTC CCA GA
7	Mouse *Mmp9*	CCA GGA TAA ACT GTA TGG CT	ACA GGA AGA GTA CTG CTT GC
8	Mouse *Dc-stamp*	ACA AAC AGT TCC AAA GCT TGC	GAC TCC TTG GGT TCC TTG CT
9	Mouse *Tnfr (TRAP) V1*	GAG TCA GAC TAA TGT CAT CTG TGG TT	ACC CCG AAA ATG GTG ATG
10	Mouse *Ctsk*	CCG AAT AAA TCT AGC ACC CTT AGT	GAA ACT TGA ACA CCC ACA TCC
11	Mouse *Caspase3*	GAG GCT GAC TTC CTG TAT GCT T	AAC CAC GAC CCG TCC TTT
12	Mouse *Anxa5*	TTG ACA TCC CGA AGC AAT G	GAC TTC AGG TCA TCC ACA AGG
13	Mouse Mapk14 (p38a) V1	CCATCTGATGTCACCAATTCA	ATTTCCAGAACCGGGGATAG
14	Mouse Mapk1 (Erk2) V1	GGATTGAAGTTGAACAGGCTCT	GAATGGCGCTTCAGCAAT
15	Mouse Mapk8 (Jnk) V1	CATACTTCATGAGCTGCAAGGT	CGCTCTTACTCCTGCTGATTG
16	Mouse Fos (c-Fos)	GGC TCT CCT GTC AAC ACA CA	GAC CAG AGT GGG CTG CAC
17	Mouse Mafb	TCCCAGGAAGAGCAGAGGAG	TTCCTTAGCTCAGCCTTGCC
